# The Behavioral Effects of Montessori Pedagogy on Children’s Psychological Development and School Learning

**DOI:** 10.3390/children9020133

**Published:** 2022-01-20

**Authors:** Edouard Gentaz, Sylvie Richard

**Affiliations:** 1 Faculty of Psychology and Educational Sciences, University of Geneva, Uni Mail, 40 Boulevard du Pont-d’Arve, 1211 Geneva 4, Switzerland; sylvie.richard@hepvs.ch; 2 Valais University of Teacher Education, Av. du Simplon 13, 1890 Saint-Maurice, Switzerland

**Keywords:** alternative pedagogy, evaluation, development, learning, language, pretend play

## Abstract

This review examines the quantitative behavioural studies that have evaluated the effects of Montessori pedagogy on children’s psychological development and school learning. The analyses of only three “Randomized Controlled Trials—RCT” studies published to date reveal varied and contradictory effects. Firstly, these findings are discussed in the light of several methodological limitations: the absence of active control groups, small sample sizes, diversity of measures, or lack of control over the implementation fidelity of both Montessori and conventional pedagogy. Secondly, these findings are discussed in the light of what Montessori pedagogy does not emphasise in its conception of development and the role of the teacher, namely the place given to language and pretend play.

## 1. Introduction

Montessori pedagogy has been very successful in recent decades [1,2]. This pedagogy has a very positive image in the media and has enthusiastic testimonials from former students and educators. Promoters of this pedagogy often rely on research results. For example, Lillard [3] discusses eight of Dr. Montessori’s major insights on how people learn and develop more optimally and how their insights are well supported by modern psychological research. There may be testimonials that have met with great editorial success and have led the general public to believe in a validated Montessori pedagogy [4,5,6]. The question is whether Montessori pedagogy has significant measurable effects on children’s cognitive and socio-emotional development and learning. The answer to this question is therefore of theoretical and practical interest to researchers and education professionals as well as to parents. Montessori pedagogy is underpinned by a theoretical corpus developed and supported by various organizations, which issue labels attesting to teaching conformity using the method, as is the case with Montessori schools. Montessori pedagogy is offered in private or/and public schools according to the country.

It is important to recall the historical and cultural context in which this pedagogy emerged to better understand its characteristics. Maria Montessori, a doctor by training, was called upon in 1902 to take care of children from an underprivileged area of Rome in Italy in the first “Children’s House” of its kind [7]. The children were not enrolled in school and were left to their own devices. Based on her observations, Montessori defends several ideas [8,9], some of which are questioned by scholars (e.g., [10]: children are driven by spontaneous curiosity and intrinsic motivation; they are generally not afraid of failure and, conversely; they require repetition until they reach acquisition perfection. Learning is thus seen as a source of satisfaction. Then, they will develop academic learning (e.g., in mathematics or reading) through a set of sensory [11] and self-corrective materials adjusted to their individual demands. It also includes the learning of social behaviours through “grace and courtesy” exercises, where each child develops body control allowing him/her to act appropriately, as well as learning congruent attitudes in social interactions and situations. The environment is organized to allow the children to act freely and independently of adults (within a precise framework), thus promoting their autonomy, their initiative, and allowing them to achieve deep concentration. The use of equipment includes movement, but it also aims to help students to channel their need for movement. Then, since the material used by the children is self-corrective, they have immediate feedback on their success, without adult intervention. The children therefore do not associate a lack of success with negative feelings. A failure serves as factual information for the child, who should be interested in persevering until he/she finds the solution independently. Formal marks, punishments or rewards are not used. Ages are blended within a class to encourage pro-social (non-competitive) interaction and mutual aid: older students may take great pride in helping younger ones (thereby allowing them to anchor their knowledge through repetition and rephrasing), and younger ones feel great satisfaction in interacting with and possibly learning from their elders.

Finally, more globally, it is necessary to recall that the Montessori pedagogy and most other alternative pedagogies were developed in the first half of the 20th century in the movement of new education, when teaching was very top-down from the teacher to the student. These alternative pedagogies put children’s needs and abilities first. Compared to this conventional top-down view of instruction, alternative pedagogies are based on a more bottom-up vision of knowledge and learning.

Currently, several structural and specific characteristics are present in schools that offer Montessori pedagogy [3]: teachers specifically trained in the pedagogy are normally recognized by Association Montessori International (AMI); a low teacher-student ratio; multi-age classes; sensory and self-correcting teaching materials; the absence of explicit, formal grades, rewards, or punishments; and the possibility for each child to have long periods of concentration.

This review does not aim to discuss in detail whether the eight principles of Montessori pedagogy correspond to current researches in developmental and learning sciences [3,5], but attempts to examine the quantitative behavioural studies that have evaluated the effects of Montessori pedagogy on children’s psychological development and school learning. However, evaluating the effects of a specific pedagogical method used in a school on the psychological development of a child is difficult and complex. This raises many methodological problems and psychosocial biases that can influence the results, such as the nature of the control group, the Pygmalion (teachers expectations about their students can influence their academic achievement of their students), and Hawthorne (results of an experience are the consequence of the teacher awareness to participate to a study in which they are evaluated, increasing their motivation) effects, etc. Moreover, studies show that participating in a new large-scale experiment generates teacher satisfaction without any systematic beneficial effect on student performance [12,13]. Before discussing the main studies that attempt to answer this question, let us examine how evaluations of pedagogy are conducted, with their principles and characteristics, their constraints in the school environment, and their limits.

## 2. The Quantitative Evaluations of Montessori Pedagogy

### 2.1. Two General Types of Scientific Approaches

In the educational field, it is traditional to consider two general types of scientific approaches. The first, called “hypothetical-deductive”, starts from theories or laws that are as general as possible, drawing predictions from them that are compared with data from experience. The second, called “explanatory”, starts from what is observed and questions its determinants by attempting to trace the causal chain. These two approaches are linked, and there is a back-and-forth flow between them. In both approaches, researchers use complex causal systems involving several explanatory factors revealed through statistical analysis. Both approaches are legitimate in the field of education [12,14].

The “hypothetico-deductive” approach is favoured in research in interventional cognitive sciences. This type of research mainly uses the so-called experimental method. Its main objective is to “administer the evidence”, i.e., to show that a factor (for example, a teaching method or learning technique) is indeed the main cause of the appearance of observed behaviour (e.g., better learning performance), “all things being equal” [15]. To acquire the certainty that this causal relationship is univocal, “interventions” (also called training, methods, or pedagogies) must be organized in the classroom to control all other factors likely to influence the observed performance (school level, socio-professional category, etc.) as much or as well as possible. This is a very difficult task given the complexity of the educational system.

### 2.2. The Measure of Effectiveness of Pedagogy: Three Levels of Evidence

To measure the effectiveness of an “intervention” or “pedagogy”, three (from A to C) levels of methodological rigour can be considered in the type of experimental protocol used by researchers [5,16]. Two main criteria can be used: presence or not of a “control group” (CG) and a random assignment of students (i.e., how students are selected and assigned to the experimental or control groups).

In this perspective, level A corresponds to studies using “intervention-only” protocols. The intervention is conducted with a single group of students, and pre- and post-intervention measures are carried out to evaluate the impact of the intervention but without comparison to a reference control group. This type of protocol makes it possible to test the “feasibility” of the intervention but prevents the interpretation of the results since no control group can ensure that students not benefitting from the intervention do not show the same evolution on the measured variables. In this review, we have chosen not to present the results of the Level A studies given their difficulties of interpretation.

Level B corresponds to studies conducting an intervention with a group of students (“experimental group—EG”) and comparing the pre- and post-intervention measurements to a control group of students with no random assignment. Studies unable to do so correspond, for example, to a student assignment guided by the teacher’s voluntary commitment to the research and, therefore, the students in his or her class. With this type of study, a distinction can also be made between the “active control group” (i.e., also benefiting from an intervention program of a different nature than that of the experimental group) and the “passive control group” (i.e., not benefiting from any intervention, or placed on a waiting list to benefit from the intervention after the study). In this case, while this type of study allows for comparison with a reference group, it does not prevent possible well-known psychosocial effects (such as positive expectations of the intervention or the placebo effect) in the group benefiting from the intervention, which may influence the measures. However, the effects of passive and active controls on cognitive interventions are in debate. Two meta-analyses to estimate their relative effect sizes showed no meaningful performance difference between passive and active controls, suggesting that current active control placebo paradigms might not be appropriately designed to reliably capture these non-specific effects or that these effects are minimal in this literature [17]. The analysis of the results of 19 level B studies (see Supplementary Materials, Table S1), comparing the effects of Montessori pedagogy on various skills at different ages, revealed that they are highly varied and contradictory [6].

Level C corresponds to studies conducting an intervention with a group of students (experimental group—EG) and comparing the pre- and post-intervention measurements to a control group (CG) of students with a random assignment of students. Studies able to carry out a random assignment are called “randomized studies or randomized controlled trials—RCT”. These studies can be “double-blind”, but this condition cannot ensure a pedagogical intervention for the teachers, in particular, or the experimenters. As with Level B, a distinction can be made between the active and passive control groups.

## 3. Main Results of the Level C Studies

Several authors have synthesized some of the studies that have evaluated the effects of Montessori pedagogy on children’s psychological development and learning [18,19,20]. We have chosen to present only and, in more detail, the only three RCT studies published to date. It is important to note that in these three studies, the experimental group is not compared to a specific curriculum (active control group) where the fidelity of implementation can be measured. For example, a comparison using a program such as “Tools of the Mind” (based on the Vygotskian perspective [21,22]) is a curriculum and teacher training program that targets the development of self-regulation while at the same time building academic skills through pretend play and social interaction. We will also indicate, when available, the impact of the effects observed. These are the differences between the mean of the two groups being compared, divided by the standard deviation of the sample. They allow us to estimate the magnitude of the differences observed. According to Cohen [23], an effect is generally considered small with a d around 0.2, medium with a d of 0.5, and large with a d of 0.8. In the educational research, Hattie [24] recommends considering the size of an effect only as a d of 0.4.

### 3.1. RCT Study 1: Lillard & Else-Quest Study (2006)

Lillard and Else-Quest [25] evaluated the effects of Montessori pedagogy on students’ performance at the end of kindergarten and the end of elementary school. The authors tested a group of 5-year-old students, 30 in Montessori classes and 25 in conventional classes; as well as another group of 12-year-old students, 29 in Montessori classes and 28 in conventional classes. A unique feature of this study is that it took advantage of a randomized system of enrolling children in a Montessori school to recruit participants. In fact, all of the students evaluated had been enrolled by their parents in the random draw to attend a Montessori school or another educational system, all of them volunteering to have their child follow Montessori pedagogy in this AMI-affiliated school. Children in the passive control group were not selected in the draw. Students were assessed with different measures of cognitive, academic, and social skills, and their performance was then compared across the board.

At age 5, Montessori students scored better on the cognitive flexibility test than conventional students (d = 0.61), but the two groups did not differ in inhibition or on the three tests assessing reasoning. Academic skills were higher for Montessori students in math problem solving (d = 0.55), reading (d = 0.44) and phonology (d = 0.63), and equivalent between the two groups in vocabulary. This lack of difference between the two groups on the vocabulary test suggests that the previous results cannot be mainly explained by the socio-economic level of the parents [26]. Students in Montessori classes also had better social cognition skills than students in conventional classes. This was observable in the theory of mind test (d = 0.61), in the proportion of responses based on the principle of justice in solving a social problem (d = 0.89), as well as on the quality of student interactions during recess. Indeed, the observations made by the experimenters revealed a greater proportion of benevolent peer-to-peer gambling (d = 0.58) and less ambiguous jostling (d = 0.72) in the Montessori group. Finally, the scores of the two groups did not differ on the wellness questionnaire.

At age 12, Montessori students wrote more creative stories (d = 0.71) with more sophisticated sentence structures (d = 0.59). However, there were no differences in spelling, punctuation, or grammar in the stories written by the children in the two groups. The 12-year-olds in the Montessori group also had better social skills (d = 0.73) and expressed better academic well-being (d = 0.54). However, there was no difference between the groups in the reasoning tasks. In addition, there were no differences between the groups in the various math and language (in reading, phonology, and vocabulary) tasks.

As this study generated a lively debate, the journal ‘*Science*’ subsequently published critical comments. Lindenfors (2007) [27] criticizes the difference in the boy-girl ratio between the two groups (the latter is lower in the Montessori group). However, studies generally show that girls tend to be more academic, applied, and therefore perform better academically. Her second criticism concerns the fact that children in the Montessori group were all educated at the same school. However, we know that there are variations in interpretation and application among different Montessori schools. Mackinnon (2007) [28], for his part, notes that the influence of classmates on test results was not considered. In fact, the students evaluated in the public system included children from the random draw and others who were not.

### 3.2. RCT Study 2: Lillard, Heise, Richey, Tong, Hart, & Bray Study (2017)

Subsequently, Lillard et al. [29] compared students’ progress in Montessori or conventional preschools, assigned according to their acceptance by lottery. The Montessori group (*n* = 70) was divided into 11 triple-level classes from two AMI-recognized Montessori public “magnet” schools. The students in the control group (*n* = 71) were divided into 71 conventional schools. Thirty of these schools were public (including 15 magnet schools, 8 conventional schools, and 7 Head Start schools), and 41 were private. The “magnet” schools are public schools that receive additional funding from the state and offer specialized education. Their admission system operates by drawing lots, and their objective is to promote social mixing by attracting students from both well-off and disadvantaged backgrounds. Head Start programs aim to prepare children from birth to 5 years old from low-income families for school by supporting their overall development. “Head Start” thus includes “Head Start” preschool programs for 3–4-year old and “Early-Early Head Start” programs for toddlers and pregnant women. The sample included a wide range of income levels and parental education, but the two groups did not differ in these demographic variables. Children were tested at least three times over the four testing sessions organized over their three years of kindergarten: once at the beginning (T1), then once at the end of each school year (T2, T3, and T4). The tests repeated those of 2006 with a few additions, such as an evaluation of perseverance in the face of effort (i.e., solving an impossible puzzle) and creativity. The authors grouped performance on the language (vocabulary and reading) and math (problem-solving and numeracy) tests into a single measure called “academic skills”. They then grouped a task assessing behavioural inhibition and visuospatial ability (figure copying) into a factor called “executive functions”. Unfortunately, the descriptive data are not reported in the article.

Analyses conducted at T1 revealed no initial differences between the groups in the different tests assessed. The authors then observed that, compared to the control group, Montessori students progressed more in the overall academic skills score (d = 0.41 at T4) and the theory of mind test (d = 0.32 at T4). In addition, they were more persistent in their efforts in T3 and T4 (65% Montessori students versus 47% control students). Montessori students at all grade levels reported on average more enjoyment of academic tasks than students in the passive control group. Nevertheless, the creativity test revealed similar results between the two groups at each assessment time. Furthermore, contrary to the results of their previous cross-sectional study, there was no difference between the two groups in the proportion of references to justice in solving a social problem and executive functions. Since the different academic skills were not examined individually, it is difficult to compare these results with those of the previous study regarding reading and mathematics learning.

### 3.3. Courtier et al. (2021)

Recently, Courtier et al. [30] conducted a randomized study that measured the language, mathematical, executive, and social skills of children at a public kindergarten in a disadvantaged socio-economic area in France. The children had been randomly assigned to classes applying Montessori pedagogy (with a measure of implementation fidelity) or conventional pedagogy. Of the 131 students tested, 53 students (27 girls/26 boys) were in Montessori classes, and 78 students (38 boys/40 girls) were in conventional classes. The authors analyzed the students’ progress over the three years of kindergarten (longitudinal study) and compared the performance of the children around 5–6 years of age (cross-sectional study).

Statistical analyses reveal an absence of difference between the two groups in the cross-sectional study at the end of the year for almost all the classic or original tests or trials: Evaluation of oral language; phonological awareness; pragmatic reading test; math problem solving; digital skills; self-regulation; short-term memory and visuospatial working memory; labyrinth test; social problem-solving test-revised; theory of mind; allocation of resources to a third party; dictator game test; feeling about school test. The results revealed better performance for the Experimental Group (EG) regarding a French reading test (decode letters, then digraphs, increasingly difficult words, and finally sentences; the test was stopped if a child was not able to read any letters or any of the digraphs). The analysis of the progress made between the two groups and between the 3–4-year-olds and the 5–6-year-olds confirms the previous results.

Regarding the literacy curriculum in both Montessori and non-Montessori public schools in France, both made use of exercises intended to develop phonological (and, in particular, phonemic) awareness, letter recognition, and knowledge of letter/sound correspondences on the understanding and use of the alphabetic principle in pre-reading kindergarten children [31,32]. These types of exercises were based on the content of the official French teaching aids. However, work on letter identities was based on visuohaptic and haptic exploration in the case of Montessori schools, whereas it was based on visual exploration alone in the case of non-Montessori schools. These results are generally contradictory with those obtained by Lillard and colleagues [25,29] in young children. More particularly concerning the beneficial effects observed in reading for the Montessori group, one explanation could be related to the contribution of multisensory and manual explorations in the rough-letter discovery exercises. Indeed, let us recall that Montessori pedagogy uses many specific, multisensory teaching materials (rough letters to be scanned with the fingers), generating several gestures and movements in young students. Numerous research studies conducted in large, conventional public-school kindergarten classes in France (in both underprivileged and socially advantaged neighbourhoods) show that the addition of manual movements to conventional exercises for discovering the alphabetical principle promotes learning to read and to decode words [33,34,35,36,37,38].

## 4. General Discussion

### 4.1. Methodological Limitations

The results reveal varied and contradictory effects of Montessori pedagogy on the psychological development of children and school learning. These results may be explained by several methodological limitations present in most of these studies, which can sometimes result from the complexity of the task.

Firstly, Montessori schools can be public or private institutions of very different sizes, ranging from small family-based care structures to schools with several hundred students. Moreover, the integration of children in Montessori schools often results from a parental choice, which indicates a family sensitivity to pedagogy. Analysis of the studies reveals that most of them are not randomized studies and, even when this is the case, the results are contradictory in 5-year-old children for certain skills.

Another argument favouring the heterogeneity of the results could be the degree of fidelity to which the Montessori method was applied. Following the observation that the fidelity of implementation of the Montessori pedagogy was not respected or was not reported in most of the previously published articles, Lillard [39] differentiated among twelve Montessori classes the so-called high fidelity Montessori classes (*n* = 36) from the so-called supplemented Montessori classes (*n* = 41). These two types of classes respected the fundamental criteria of Montessori pedagogy, such as multi-aging, individualization of teaching, or lack of rewards. Nevertheless, the supplemented Montessori classes offered non-Montessori materials (e.g., handicrafts, puzzles, legos, workbooks) and the interruption of working time by special lessons (e.g., foreign languages, music) two to three times a week. Supplemented Montessori classes also sometimes involved a second teacher in the class. Thus, 95–100% of the students used Montessori materials in the three so-called high-fidelity classes, compared to 38–56% in the nine supplemented classes. Employing the tests used in 2006, the researchers observed greater progress in the high-fidelity group for executive functions, reading, vocabulary, and mathematics, but not for the other tests of social skills and mind theory (in which the Montessori material would not be involved according to the authors).

In this vein, it is important to recall that the three selected RCT studies do not include a fully active control group with an alternative pedagogy that would be measurable on the level of its implementation. This aspect is also another limitation that should be considered in future research evaluating the effectiveness of an integrated curriculum in the daily classroom reflecting a certain type of pedagogy (e.g., [36,40,41,42]).

In summary, the criterion of school selection, the degree of implementation of the pedagogy, and the necessity to include an active control group, thus appears crucial when one wishes to study its effects on children. Moreover, while recent studies indicate the degree of implementation of Montessori pedagogy, none measure the degree of fidelity of conventional schools to the official curriculum or of schools to each other in that same way.

Then, Courtier (2019) [18] highlighted the variations in the choice of analyses performed in different studies: some variables are transformed into average scores based on the sample scores, some measures are at times studied separately, and others are studied together. For example, measures of “language skills” combine measures of vocabulary and reading skills [43], while “academic skills” combine both language and mathematical skills [29]. These variations are likely to produce differences in the statistical results, not due to the data but to the transformations performed on the data before analyzing them.

A final limitation is the publication bias of this type of study. This bias means that the majority of the published studies show an effect, which potentially prevents the dissemination of non-significant results. This publication bias mechanically leads to the over-representation of false positives and the overestimation of effect sizes in meta-analyses [44]. Moreover, this bias may partly explain the relative reproducibility (reliability) of the results. Recall that in 2015, a group of researchers attempted to replicate about 100 studies (experimental or correlational) published in peer-reviewed journals of good quality and reproduced the original results of only 50% of these studies [45].

Finally, if we analyse the latest studies published very recently, most beneficial effects are observed when researchers test students from high socio-economic backgrounds voluntarily enrolled in private schools (Denervaud et al. [46,47] and in Montessori private group tested by Courtier et al. [30]). In contrast, almost no effect (except in reading) is observed when students are randomly assigned to Montessori classes and come from public schools in underprivileged neighbourhoods (in both the Montessori public group and the conventional public group tested by Courtier et al. [30]).

Therefore, it seems crucial to take these factors into account to understand past results and implement future studies. These future researches may be addressed by considering the assessment of implementation fidelity through systematic observations in the classrooms by trained external observers, ensuring the flexibility of analyses using preregistration to share the specificities of the research in a public field before conducting the study as Courtier et al. [30] did, in this sense the difficulty of publishing null results can be addressed by registered reports.

### 4.2. The Place of Language and Pretend Play in the Montessori Method

The fact that the results obtained in this article do not make it possible to affirm that this type of pedagogy prevails over a more “conventional” pedagogy leads us to believe that other factors linked to the vision of child development defended by Montessori need to be considered. This leads us to consider a second broad category of limits related to Montessori’s conception of development and the role played by the teacher. We will focus on two specific aspects, namely, the role of language and pretend-play in development.

For Montessori, language is a “byproduct” of knowledge. It reflects what the child has already concluded on their own. For instance, learning words to describe different colours is a consequence of training the visual ability to distinguish them [48]. In contrast, in other theories of development, such as that advocated by Vygotsky, language facilitates the sharing of experiences necessary for constructing cognitive processes. It is also an important skill for appropriating the mental tools of others [48]. If we take the previous example of learning words for two colours, in the Vygotskian perspective, the acquisition of two different words helps the child to see that there is a difference between the two colours. Language is also seen as an essential component in the child’s rational activity. In this perspective, how the teacher uses language in the daily classroom is also different. In Montessori pedagogy, “The main role of the teacher is to direct the child’s psychic activities and patiently await their spontaneous manifestation” (freely translated from De Serio, 2013, p. 52, [49]). In other words, the teacher directs the child’s psychic activities (being the mediator between the child and the material) without interfering with the spontaneous maturation of child development [49]. In the Vygotskian perspective, it is the opposite: with the help and collaboration of the teacher (e.g., by putting the child on the path asking questions, by giving them the beginning of the solution, by showing, etc.), children can solve more difficult problems that they would not have been able to solve alone, which is represented by the “proximal zone of development” (Vygotsky, 1997, p. 351, [50]). In this conception, the nature of the processes of development of the higher psychic functions is purely social. By contrast, in the Montessori method, “didactic material” plays a central role, because it contains within itself the control of errors and which makes auto-education possible for each child” (Montessori, 1912, p. 372, [8]). The role played by the teacher is to watch children, make observations, show how to use the structured material, and represent a “silent presence” in the daily classroom.

The second factor not considered an important activity for child development is pretend-play. Vygotsky (1933/2016) [51] defined make-believe play as an activity in which the child uses their imagination to create fictional situations, selects, adopts, and interprets roles, and applies rules that correspond to the chosen roles and scenario. In the Vygotskian perspective, pretend play represents a “leading activity” in children aged 3 to 7 years, which means the most favourable activity to generate developmental gains specific to this age [52]. In contrast, “Pretending has no place in Montessori education, […]. Children in a Montessori classroom might want desperately to play house with the little broom and mop set (as I did as a child), but the teachers gently direct them to other, real work, like actually mopping the floor” [53] (Lillard, 2013, p. 173). Montessori thought that pretend-play should be avoided in favour of more “productive” activities. She considered that a child’s imagination was a product of mind immaturity, and in consequence, the child’s mind should be enriched with real activities directed towards real goals (a concrete example is “Practical Life Activities”, such as cleaning the table or preparing a snack) [54]. As indicated by Montessori (1912): “The child who left the game in his eagerness for knowledge, has revealed himself as a true son of that humanity which has been throughout centuries the creator of scientific and civil progress” [8] (p. 372). Yet, there is growing evidence in the existing literature of correlational and interventional studies demonstrating the benefits of this form of play for children’s cognitive and socio-emotional development. For example, in a recent interventional study, Richard, Baud-Bovy, Clerc-Georgy, and Gentaz (2021) [55] showed an improvement in children’s ability to recognize emotions and associate facial expressions of emotion to the correct emotional label in 5- to 6-year-old children. Five teachers implemented a program based on pretend-play, including structured teaching/learning periods centered on socio-emotional competencies (e. g., discussions with the whole group of children on how to recognize basic emotions). These more structured times were systematically followed by reinvestment periods of this knowledge in the context of pretend play (e.g., mimicking emotions, playing emotions-generating social situations, and discussing the emotions expressed and what triggered them in their play). The effect of social pretend play on inhibitory control was also shown in 132 preschoolers (Age = 53 months) [56]. The proportion of social pretending during free-play was associated with the growth of inhibitory control throughout the school year. Social pretend play was the only predictor of gains in inhibitory control. Interestingly, solitary pretending did not predict changes in inhibitory control skills, nor did social play alone (e.g., interacting, or conversing with peers).

In conclusion, many features in the Montessori pedagogy positively take children’s developmental needs into account and should be an integral part of teachers’ working modalities in so-called “conventional” public schools (such as the possibility for each child to be in multi-age classes; to have an absence of explicit, formal grades, rewards, or punishments; and a low teacher-student ratio). However, Montessori’s view of the role played by language as well as by pretend-play should be analysed more carefully and could also partly explain the contrasting results of the different studies highlighted in this article.

Finally, it is critical not to ignore the role played by parents in their childrens’ global development (which is different in the two first RCTs studies compared to Courtier et al., study). The fact that children in Montessori pedagogy are globally at least as competent as other children in more conventional pedagogy is likely due to the parent’s conception of education finality (e.g., the importance of the development of autonomy, the necessity to take into account the child’s specific needs, and learning rhythm). Maybe, these parents are already sensitive to the importance of scaffolding their children’s language skills from birth, allowing more time for certain forms of play, and maybe are more concerned about their child’s academic future than other parents who are less inclined or aware of the importance of these stimulations. They may also have at least partially compensated the results obtained on some measures. In the two first RCTs studies described above, the recruitment process leads us to believe that the children selected are likely to have parents who are maybe more academically involved and supportive. In the first RCT study, all parents volunteered to have their child follow Montessori pedagogy in an AMI-affiliated school. In the second RCT study, parents “had submitted a lottery application (…) selecting one of the two Montessori schools as their first of five school choices” [29] (Lillard et al., 2017). The effect of daily parental language and play stimulations, as well as their academic involvement, should be considered in future studies, as it (academic involvement) influences academic achievement [57].

## Supplementary Materials

The following supporting information can be downloaded at: https://www.mdpi.com/article/10.3390/children9020133/s1, Table S1: Summary of the main level B studies comparing the effects of Montessori pedagogy on various skills at different ages (translated and adapted from [6]).
