# Supplementary material for: The Behavioral Effects of Montessori Pedagogy on Children’s Psychological Development and School Learning
Source: Children (Basel). 2022 Jan 20;9(2):133. doi: 10.3390/children9020133 (PMC8870616; doi:10.3390/children9020133)
Supplement: Supplementary file 1 [file children-09-00133-s001.zip › children-1484114-supplementary.pdf]

Supplementary Materials

Table S1: Summary of the main level B studies comparing the effects of Montessori pedagogy on various skills at different ages (translated and adapted from Gentaz, Rivier and Richard [1].

**Studies**<br>Age<br>Measures	**Montessori group - EG**	**Control Groups - CG**	**Main results**	**Comments**
Lopata and Pasnak [2]<br>8 and 12 years<br>Math and Language	N=88 students	N=114 in « magnet » schools with structured lessons (CG 1)<br>N=175 in “magnet” schools with free instruction (CG 2)<br>N= 166 in neighbourhood schools (CG 3)	At age 8<br>Math: EG = CG 1; EG > CG 2 (d=0.6)<br>EG < CG 3 (d=0.37)<br>Language: EG = CG 1 = CG 2 = CG 3<br>At age 12<br>Math: EG = CG 1 = CG 2 = CG 3<br>Language: EG < CG 1 (d=0.77)<br>EG = CG 2; EG < CG 3 (d=0.59)	The parents chose to send their children to Montessori or “magnet” schools
Manner [3]	N=30	N=30	Math: EG = CG at age 8, 9 and 10	

8, 9 and 10 years<br>Math /Reading			Reading: EG > CG at age 8, 9 and 10
Mallett and Schroeder [4]<br>6 to 10 years<br>Math and Reading	N=518	N=517	At age 6, 7 and 8:<br>Math: EG = CG Reading: EG = CG<br>At age 9 and 10:<br>Math: EG > CG Reading: EG > CG
Brown and Lewis [5]<br>8 years<br>Maths and Reading	N=335	N=1348 in conventional and "magnet" schools	Math: EG = CG<br>Reading: EG > CG
Mix, *et al.* [6]<br>5 and 7 years<br>Three math tests	N=34	N=34	At age 5: The decimal system: EG = CG<br>Division: EG = CG<br>Number line: EG = CG<br>At age 7: The decimal system: EG > CG<br>Division: EG > CG<br>Number line: EG = CG
Byun, *et al.* [7]<br>4 years	N=164	N=167	During school: EG > CG<br>After school: EG > CG

Locomotor movement				
Bhatia*, et al.* [8]<br>5 years<br>Fine motor skills	N=50	N=50	Precision of the thumb-index pinch<br>EG > CG (d=0.53)<br>Pinch speed<br>EG > CG (d=0.37)	Private Montessori schools and public conventional schools
Bagby*, et al.* [9]<br>9, 10 and 11 years<br>Executive functions with questionnaires	N=33	N=39 in conventional schools (CG 1)<br>N=40 in Christian schools (CG 2)	Evaluations by parents<br>EG = CG 1 = CG 2<br>Evaluations by teachers<br>EG and CG 1 > CG 2	
Besançon and Lubart [10]<br>5 to 12 years<br>Creativity	N=40	N=51; Freinet pedagogy (CG 1)<br>N=119 in conventional schools (CG 2)	At all ages<br>EG > CG 1 and CG 2	
Kirkham and Kidd [11]<br>5 to 9 years<br>Creativity based on drawings	N=20	N=20; Steiner pedagogy (CG 1)<br>N=20 in conventional schools (CG 2)	EG < CG 1<br>EG = CG 2	Higher socio-economic level for Montessori parents

Cox and Rowlands [12]<br>5 and 7 years<br>Producing three types of drawings	N=20	N=20; Steiner pedagogy (CG 1)<br>N=20 in conventional schools (CG 2)	Free drawing from observation:<br>EG < CG 1 and EG = CG 2<br>Drawings of scenes:<br>EG < CG 1 and CG 2
Rathunde and Csikszentmihalyi [13]<br>12 to 14 years<br>Affective characteristics through self-report questionnaires according to activity	N=135	N=151	For academic activities<br>EG > CG<br>For non-academic activities<br>EG = CG
Dhiksha and Shivakumara [14]<br>15 years<br>Emotional intelligence through self-report questionnaires	N=549	N = 533	EG > CG
Denervaud, *et al.* [15]<br>5 to 12 years<br>Emotion recognition	N=28	N=29	EG > CG

Taggart*, et al.* [16]<br>3 to 6 years<br>Preference for pretend play or realistic play	N=54	N=62	EG > CG	
Lillard [17]<br>3 and 6 years<br>Academic, social and cognitive skills	N=36; high fidelity (EG 1)<br>N=95; medium fidelity (EG 2)	N=41	Executive functions, EG 1 > EG 2 and CG<br>Vocabulary: EG 1 > EG 2; EG 1 = CG<br>Math: EG 1 > EG 2; EG 1 = CG<br>Social skills: EG 1 = EG 2 and EG 1 > CG<br>Theory of mind: EG 1 = EG 2 = CG	
Ansari and Winsler [18]<br>4 to 5 years<br>Various skills	N=770	N=12975 (high scope program; half guided, half independent instruction	EG = CG	
Denervaud*, et al.* [19]<br>6 and 10 years<br>Various abilities	N=99<br>(30 6-year-olds and 69 10-year-olds)	N=102<br>(28 6-year-olds and 74 10-year-olds)	Language, Math, creativity, working memory: EG > CG<br>Attentional Selectivity, cognitive flexibility: EG = CG	Average effect size

Dohrmann, *et al.* [20]	N=201 had gone to a	N=201 had gone to a	Global score: EG = CG
16-18 years	Montessori school	conventional school	Math/Science: EG > CG (d =0.12)
School tests	from age 3 to 12	from age 3 to 12	Language/Social Sciences: EG =CG

References:

1. Gentaz, E.; Richard, S.; Rivier, C. Evaluation des effets de la pédagogie Montessori sur le développement psychologique de l'enfant et les apprentissages. In *L'ecole autrement? les pedagogies alternatives en debat* Darbellay, F., Moody, Z., Louviot, M., Eds. Alphil - Presses Universitaires Suisse: Neuchatel, Suisse, 2021; pp. 205-227.
2. Lopata, D.; Pasnak, R. Accelerated conservation acquisition and IQ gains by blind children. *Genetic Psychological Monographs* **1976**, *93*, 3-25.
3. Manner, J. Montessori vs. Traditional Education in the Public Sector : Seeking Appropriate Comparisons of Academic Achievement. *Forum on Public Policy Online* **2007**, *2*.
4. Mallett, J.; Schroeder, J. Achievement outcomes : A comparison of montessori and non-Montessori public elementary school students. *Journal of Elementary Education* **2015**, *25*, 39-53.
5. Brown, K.; Lewis, C. A comparison of reading and math achievement for African American third grade students in Montessori and other magnet schools. *The Journal of Negro Education* **2017**, *86*, 439-448.
6. Mix, K.; Smith, L.; Stockton, J.; Cheng, Y.-L.; Barterian, J. Grounding the symbols for place value : Evidence from training and long-term exposure to base-10 models. *Journal of Cognition and Development* **2017**, *18*, 129-151.
7. Byun, W.; Blair, S.; Pate, R. Objectively measured sedentary behavior in preschool children : Comparison between Montessori and traditional preschools. *International Journal of Behavioral Nutrition and Physical Activity* **2013**, *10*, 2.
8. Bhatia, P.; Davis, A.; Shamas-Brandt, E. Educational gymnastics : The effectiveness of Montessori practical life activities in developing fine motor skills in kindergartners. *Early Education and Development* **2015**, *26*, 594-607.
9. Bagby, J.; Barnard-Brak, L.; Sulak, T.; Jones, N.; Walter, M. The effects of environment on children's executive function : A study of three private schools. *Journal of Research in Childhood Education* **2012**, *26*, 418-426.
10. Besançon, M.; Lubart, T. Differences in the development of creative competencies in children schooled in diverse learning environments. *Learning and Individual Differences* **2008**, *18*, 381-389.
11. Kirkham, J.; Kidd, E. The Effect of Steiner, Montessori, and national curriculum education upon children's pretence and creativity. *The Journal of Creative Behavior* **2017**, *51*, 20-34.

12. Cox, M.; Rowlands, A. The effect of three different educational approaches on children's drawing ability : Steiner, Montessori and traditional. *British Journal of Educational Psychology* **2000**, *70*, 485-503.
13. Rathunde, K.; Csikszentmihalyi, M. The social context of middle school : Teachers, friends, and activities in Montessori and traditional school environments. *The Elementary School Journal* **2005**, *106*, 59-67.
14. Dhiksha, J.; Shivakumara, K. The effect of montessori and traditional methods of education on emotional intelligence of children. *European Journal of Education Studies* **2017**, *3*.
15. Denervaud, S.; Mumenthaler, C.; Gentaz, E.; Sander, D. Emotion recognition development : Preliminary evidence for an effect of school pedagogical practices. *Learning and Instruction* **2020**, *69*.
16. Taggart, J.; Heise, M.; Lillard, A. The real thing : Preschoolers prefer actual activities to pretend ones. *Developmental Science.* **2018**, *21*.
17. Lillard, A. Preschool children's development in classic Montessori, supplemented Montessori, and conventional programs. *Journal of School Psychology* **2012**, *50*, 379-401.
18. Ansari, A.; Winsler, A. Montessori public school pre-K programs and the school readiness of low-income Black and Latino children. *Journal of Educational Psychology* **2014**, *106*, 1066-1079.
19. Denervaud, S.; Knebel, J.; Hagmann, P.; Gentaz, E. Beyond executive functions, creativity skills benefit academic outcomes: Insights from Montessori education. *PLoS ONE* **2019**, *14(11): e0225319*.
20. Dohrmann, K.; Nishida, T.; Gartner, A.; Lipsky, D.; Grimm, K. High school outcomes for students in a public Montessori program. *Journal of Research in Childhood Education* **2007**, *22*, 205.
